# Baby-Led Introduction to SolidS (BLISS) study: a randomised controlled trial of a baby-led approach to complementary feeding

**DOI:** 10.1186/s12887-015-0491-8

**Published:** 2015-11-12

**Authors:** Lisa Daniels, Anne-Louise M. Heath, Sheila M. Williams, Sonya L. Cameron, Elizabeth A. Fleming, Barry J. Taylor, Ben J. Wheeler, Rosalind S. Gibson, Rachael W. Taylor

**Affiliations:** Department of Human Nutrition, University of Otago, PO Box 56, Dunedin, 9054 New Zealand; Department of Medicine, University of Otago, PO Box 56, Dunedin, 9054 New Zealand; Department of Preventive and Social Medicine, Dunedin School of Medicine, University of Otago, PO Box 56, Dunedin, 9054 New Zealand; Department of Women’s and Children’s Health, Dunedin School of Medicine, University of Otago, PO Box 56, Dunedin, 9054 New Zealand; Edgar Diabetes and Obesity Research Centre, Dunedin School of Medicine, University of Otago, PO Box 65, Dunedin, 9054 New Zealand

**Keywords:** Baby-Led Weaning, Complementary feeding, Energy self-regulation, Childhood obesity, Iron deficiency

## Abstract

**Background:**

In 2002, the World Health Organization recommended that the age for starting complementary feeding should be changed from 4 to 6 months of age to 6 months. Although this change in age has generated substantial debate, surprisingly little attention has been paid to whether advice on *how* to introduce complementary foods should also be changed. It has been proposed that by 6 months of age most infants will have developed sufficient motor skills to be able to feed themselves rather than needing to be spoon-fed by an adult. This has the potential to predispose infants to better growth by fostering better energy self-regulation, however no randomised controlled trials have been conducted to determine the benefits and risks of such a “baby-led” approach to complementary feeding. This is of particular interest given the widespread use of “Baby-Led Weaning” by parents internationally.

**Methods/Design:**

The Baby-Led Introduction to SolidS (BLISS) study aims to assess the efficacy and acceptability of a modified version of Baby-Led Weaning that has been altered to address potential concerns with iron status, choking and growth faltering. The BLISS study will recruit 200 families from Dunedin, New Zealand, who book into the region’s only maternity hospital. Parents will be randomised into an intervention (BLISS) or control group for a 12-month intervention with further follow-up at 24 months of age. Both groups will receive the standard Well Child care provided to all parents in New Zealand. The intervention group will receive additional parent contacts (*n* = 8) for support and education on BLISS from before birth to 12 months of age. Outcomes of interest include body mass index at 12 months of age (primary outcome), energy self-regulation, iron and zinc intake and status, diet quality, choking, growth faltering and acceptability to parents.

**Discussion:**

This study is expected to provide insight into the feasibility of a baby-led approach to complementary feeding and the extent to which this method of feeding affects infant body weight, diet quality and iron and zinc status. Results of this study will provide important information for health care professionals, parents and health policy makers.

**Trial registration:**

Australian New Zealand Clinical Trials Registry ACTRN12612001133820.

## Background

In 2002, the World Health Organization (WHO) recommended that the age when complementary feeding, or the introduction of “solid” foods, should start should be changed from 4 to 6 months to 6 months of age [1, 2]. This change was a consequence of the WHO recommending an extension to the exclusive breastfeeding phase from 4 to 6 months to 6 months (180 days) of age [3]. By 6 months of age, the infant’s renal function, digestive function and oral motor skills (i.e. chewing and swallowing) have developed enough to manage solid foods [4]. Furthermore, by this age complementary feeding is needed to ‘complement’ the nutrients and energy provided by breast milk to ensure appropriate growth and development [3]. Although there has been considerable debate about this change in the age when complementary feeding should be initiated [5–7], there has been surprisingly little attention paid to whether advice on *how* to introduce complementary foods should also be changed given the substantial development in gross, fine and oral motor skills that occurs between 4 and 6 months of age.

Traditionally, complementary feeding has been based on graduated exposure to solid foods with different textures [8–10]. This means that infants are given puréed foods before progressing to mashed and chopped foods, with ‘finger’ foods not contributing a large part of the diet until later in the complementary feeding period (typically around 8–9 months of age). As outlined in Table 1, this advice has changed little in response to the change in the recommended age for introducing complementary foods, despite marked changes in physical development between 4 and 6 months of age. Puréed foods may have been necessary at 4 months because infants have a limited ability to chew at this age and most are not able to sit unsupported [11]. However, by 6–7 months of age most infants are able to chew, sit unsupported and bring foods to their mouth [11], suggesting that a gradual transition from purées to finger foods may now not be necessary [12]. If this is indeed the case, then both the types of foods offered, and the role of parents in infant feeding, may be altered and this may have important implications for infant health outcomes including obesity, nutritional status and choking risk.

**Table 1:** Appropriate textures for complementary feeding according to current recommendations in New Zealand [8], United States of America [9], and Australia [10]

Approximate age	Appropriate texture
0 – 6 months	Liquid
6 – 7 months	Puréed
7 – 8 months	Mashed and “Finger”^a^ foods
8 – 12 months	Chopped
12 – 24 months	“Family”^b^ foods

^a^ Finger foods are foods that can be picked up by the child and eaten “with the fingers”

^b^ Family foods are foods that are eaten by the rest of the family, in the form that they are eaten by the rest of the family

### Baby-Led Weaning

Baby-Led Weaning (BLW) differs from the traditional approach to complementary feeding because the infant is encouraged to feed themselves *all* their foods from the beginning of the complementary feeding period [12]. While most countries recommend that finger foods are included in the complementary feeding period, even from as early as 6 months of age in the United Kingdom (UK) [8, 10, 13, 14], they generally only represent a small component of the complementary feeding diet, particularly in the first few months. In contrast, parents following BLW choose a range of foods to offer their infant and the infant decides which of the foods to eat, how much and at what pace they will eat them [12]. The key features of BLW are [12, 15]:

- Milk feeding – the infant will ideally be exclusively breastfed until 6 months of age, although it is acknowledged that some infants will be formula fed. When complementary feeding starts (once the infant is ready, at around 6 months of age) the infant continues to receive milk feeds (breast milk or infant formula) on demand.
- Baby-led – the infant self-feeds from the beginning of the complementary feeding period. Generally speaking puréed foods are not eaten because they need to be spoon-fed and therefore fed by someone other than the infant. Some families may offer the child utensils so that they can feed themselves purées or foods with a thin consistency (e.g., yoghurt and custard) but this is unlikely in the first few months for developmental reasons.
- Family foods – the infant is offered the same foods as the family but as finger food that is large enough for them to pick up. These pieces can get smaller with increasing developmental age.
- Mealtimes – the family eats together at mealtimes.

Although BLW has received considerable attention in both the scientific literature [11, 12, 16–22] and the lay media, the New Zealand Ministry of Health does not currently recommend BLW as an alternative to current complementary feeding advice because of the paucity of research on the topic [23]. Although agencies such as the United Kingdom Department of Health [13] and Health Canada [14] suggest that finger foods can be offered as *part* of the diet from the beginning of complementary feeding at 6 months of age, they do not recommend a baby-led approach in which the entire diet is self-fed.

### Potential advantages of Baby-Led Weaning

A number of potential advantages of BLW have been proposed, including: a lower risk of obesity, as a result of better energy self-regulation; better diet quality; favourable effects on parental feeding practices; and more highly developed motor skills [24].

#### Lower risk of obesity

One potential advantage proposed by advocates of Baby-Led Weaning is that it may encourage improved energy self-regulation [12], defined as “the capacity to adjust the quantity eaten according to the physiological needs of the consumer” [25]. In turn, this is expected to lower the risk of obesity. Advocates propose that the milk-only diet that infants consume from birth allows them to be in control of when and how much they consume, particularly if they are breastfed on demand. However, when complementary foods are introduced using the traditional spoon feeding approach, the parent has much more control and is likely to encourage the child to eat until they have consumed an amount of food that the parent, rather than the child, considers is “enough” [24]. By contrast, BLW encourages the infant to be in control of the amount eaten and it is suggested that this may support the responsiveness to internal hunger and satiety cues, leading to better energy self-regulation [16, 23, 26]. There is increasing evidence that better energy self-regulation is associated with a lower risk of obesity [27].

To date, only two studies have investigated rates of obesity in infants following BLW [17, 21]. Brown and Lee [21] found no association between the complementary feeding method (BLW or spoon feeding) and parentally reported infant weight at 6 months in a large (*n* = 652) cross-sectional study. However, when they measured a subset (*n* = 298 participants at 18–24 months of age who consented to follow-up contact and met inclusion criteria) they found that toddlers who had followed BLW as infants had significantly lower mean body weight (by 1.07 kg), than those who had followed a traditional ‘parent-led’ spoon feeding approach [28]. Moreover, the infants who had followed BLW were reported by their parent to be significantly more satiety-responsive (able to regulate intake of food in relation to satiety) and significantly less food-responsive (eating in response to food stimuli regardless of hunger), than their traditionally fed peers [28]. Similarly, Townsend and Pitchford [17] reported significantly lower Body Mass Index (BMI) and incidence of obesity in children at 20–78 months who had followed BLW compared to those who had been spoon-fed. However, different methods were used to recruit the BLW and spoon-fed participants and standardized procedures for measuring body weight were only used in the spoon-fed group, making these results difficult to interpret.

These initial studies are intriguing and suggest that a baby-led method of complementary feeding may help to address the growing obesity problem worldwide [23]. However, it is not possible to conclude from these cross-sectional studies that BLW itself was responsible for differences in body weight, or energy self-regulation, particularly because parents who follow BLW have been shown to differ from parents following traditional methods of complementary feeding in demographic, psychological and parenting characteristics known to also be associated with body weight [19, 21]. Only a randomised controlled trial can confirm whether a beneficial relationship exists between infant self-feeding and body weight.

#### Better diet quality

While it is often assumed that infants following BLW will consume diets of better quality, there are very limited dietary data from infants following BLW. Rowan and Harris [22] used three day diet records to assess foods eaten by parents whose infants were following BLW, in order to determine whether BLW influenced the parents’ food intake. Although the authors reported that a wide range of foods were *offered* to the infants, the infants’ actual nutrient *intake* was not determined. Furthermore, the study was a pilot study so was very small (*n* = 10 participants).

It is possible that BLW may promote acceptance of a wider range of foods as a result of early exposure to a range of different tastes and textures from a variety of foods [15], but this has not yet been formally investigated. One cross-sectional study found that infants who were mostly being fed using the BLW method were more likely to be consuming family foods (*p* = 0.018), were more likely to begin this at the start of complementary feeding (*p* <0.001) and were less likely to be given commercial infant foods (*p* = 0.002), compared with infants whose parents were following a more traditional ‘parent-led’ spoon feeding approach [19]. Family foods would be expected to be more varied in taste and texture than the foods offered at the start of complementary feeding (predominantly puréed fruit, vegetables or cereal). However, a positive effect of family meals on the infant’s diet relies on the family having healthy foods that are also suitable for the infant [18].

#### Favourable effects on parental feeding practices

One area of recent interest concerns the role that parental feeding practices may play in promoting excessive weight gain in very young children [27, 29, 30]. Exerting greater control over a young child’s food intake is thought to negatively impact on the child’s ability to regulate their energy intake. Certainly parents who follow BLW have reported lower levels of restriction, pressure to eat and monitoring of the child’s food intake and are less concerned about the child’s body weight [21]. However, it is not clear whether parents with these characteristics are more likely to choose, or to persist with BLW, or whether BLW encourages the development of these characteristics. Longitudinal or intervention studies are needed to help determine the direction of this association.

#### More highly developed motor skills

Carruth and Skinner [31] have suggested that some feeding behaviours may be achieved later by children whose parents limit their opportunities to explore during feeding time, perhaps because of concerns about mess and spills [31]. They suggest that some parents may need more encouragement to allow their child to engage in activities relating to feeding in order to help their child develop feeding skills [31]. The ability of children to learn to self-feed depends, therefore, not so much on the innate development of fine, gross and oral motor skills, but on the opportunity to develop these skills through applying them repeatedly [31]. We hypothesize that a baby-led approach to complementary feeding would provide an infant with greater opportunities (both in frequency and duration) to develop their gross and fine motor skills.

### Potential disadvantages of Baby-Led Weaning

Several concerns have also been raised about this alternative approach to complementary feeding; namely that BLW could increase the risk of iron deficiency, choking and growth faltering in infants [12, 18, 23].

#### Iron deficiency

It is important that complementary foods high in iron are introduced at 6 months of age in order to maintain adequate iron status [8]. Iron deficiency, a common nutritional deficiency globally, can lead to iron deficiency anaemia which is associated with delays in cognitive function that may not be reversible [32].

Unfortunately, the most common ‘first foods’ introduced to infants, including fruits and vegetables, are naturally low in iron. Iron-fortified infant cereals can be an important source of iron for this age group [33], however they are not likely to be consumed by infants following BLW because infants of this age will find it difficult to feed themselves this relatively liquid food. Iron rich foods such as red meat may be served in forms that can be easily picked up by a 6 month old so may be useful foods to feed from the start of complementary feeding [19, 34], as long as they are not avoided due to parental concerns about choking. To date, no studies have examined either the iron intake or iron status of children following a baby-led approach to complementary feeding.

#### Choking

Choking can easily occur in infants learning to eat as they are experimenting with moving foods around the mouth, biting and chewing and they also have small air passages [8]. The potential for choking to occur when following BLW is of considerable concern amongst health professionals and parents [18]. There are currently very few data on the rates of choking during the complementary feeding period, and no data on choking in infants following BLW. However, Cameron et al. [18] found 30 % of a group of women using BLW with their infants (*n* = 20) reported an episode of choking. This was caused by consumption of raw apple in all cases where the mother was able to recall the food responsible.

#### Growth faltering

In the first year of life, the majority of infants receive most of their energy from breast milk (or infant formula) [35]. However, complementary foods are an important source of energy and many nutrients in the second 6 months of life [8, 35]. Some health professionals have expressed concern that infants following BLW may be at increased risk of growth faltering, based on the assumption that not all infants will have the motor skills, or motivation, to feed themselves the amount of food they require, and that many of the first foods offered will be low in energy [15]. However, only two cross-sectional studies appear to have examined growth in infants following BLW [17, 28]. Townsend and Pitchford [17] found an association between weaning style and infants classified as underweight. Infants whose parents reported having followed BLW had a higher prevalence of underweight (4.8 %) than infants whose parents reported following a spoon-fed approach to complementary feeding (0 %) [17]. Similarly, Brown et al. [28] found a higher prevalence of underweight in their BLW group (5.4 %) compared with their standard complementary feeding group (2.5 %). However, both studies were limited by their cross-sectional design, the small numbers of participants classified as underweight (*n* = 3-11), parents retrospectively self-reporting the type of complementary feeding method they had used and recruitment of BLW and control groups from different sources.

#### Other potential disadvantages

Because of the limited research conducted on BLW, it is not known whether BLW poses any risks for nutrients other than iron (which is discussed above). In particular, zinc is found in limited amounts in foods that may be used as first foods in BLW, such as fruit and vegetables, because they are easy to self-feed. Poorer zinc status could have implications for growth, motor and cognitive development, and immune function [36].

Concerns have also been expressed that family foods may not always be suitable for infants if the family is consuming meals high in sugar or salt [15], both of which are inappropriate for infants [8]. Family foods offered to infants must be suitable, both because of the immediate risk to infant health and because the infant may become accustomed to salt and sugar tastes, potentially predisposing them to poorer diets in later life and therefore poorer health outcomes in adulthood.

### Summary

Although a baby-led approach to complementary feeding appears to have many potential advantages, there are still many unanswered questions, in particular:

- What impact does it have on the growth and development of infants?
- Do infants consume foods containing sufficient micronutrients?
- Does it affect the quality of infants’ diets overall?
- Does it alter parental feeding behaviours?
- Is it safe?
- Is this alternative method of complementary feeding acceptable to parents?

There are currently very limited longitudinal data and no randomised controlled trials investigating a baby-led approach to complementary feeding. A randomised controlled trial is urgently needed in order to determine the answers to these questions, both because an increasing number of parents are choosing to follow BLW, and because, if a baby-led approach to complementary feeding proves to be protective against excess weight gain in infancy, it is essential to know whether it is both safe for infants and acceptable for parents, before it can be advocated as a public health intervention.

### Aims and objectives

The aim of the BLISS study is to determine whether a novel approach to complementary feeding using foods that an infant can feed themselves - ‘Baby-Led Introduction to SolidS’ (BLISS) - prevents overweight in young children by improving energy self-regulation, without increasing the risk of iron deficiency, choking and growth faltering.

The primary objective of the BLISS study is to determine whether BLISS improves weight status (BMI-for-age z-score) at 12 months of age. Secondary objectives are to determine whether BLISS:

(i) improves energy self-regulation at 12 months
(ii) improves iron and zinc intake and status at 12 months
(iii) improves diet quality at 7 and 12 months
(iv) impacts favourably on parental feeding behaviours at 12 months
(v) results in more highly developed motor skills at 6, 8 and 12 months
(vi) is an acceptable option for parents (mess, overall acceptability, adherence) at 7–9 months; or
(vii) is not an acceptable approach to infant feeding because it increases the risk of choking or growth faltering between 6 and 12 months of age
(viii) improves weight status, energy self-regulation, diet quality, parental feeding behaviours and infant motor skills at follow up at 24 months of age.

## Methods/Design

### Study design

**Fig. 1:**
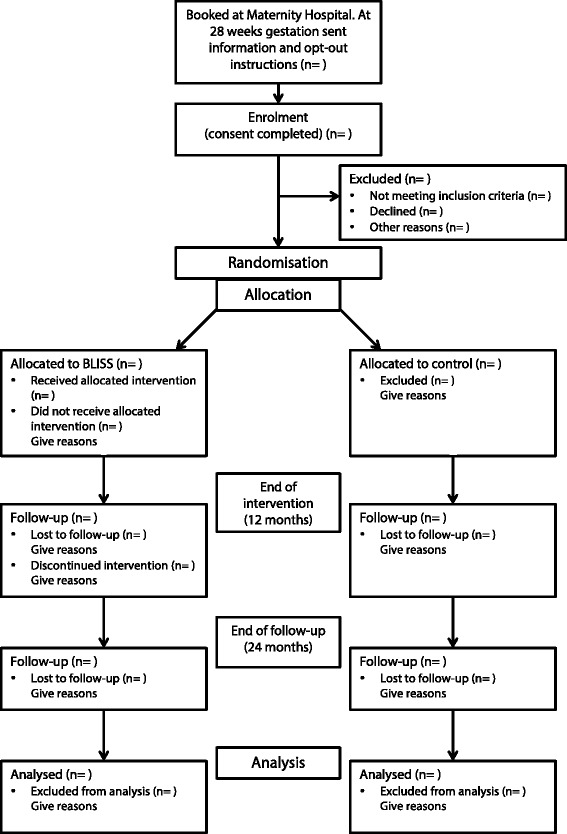
Study design

The Baby-Led Introduction to SolidS (BLISS) study is a 2-arm randomised controlled trial (Fig. 1), commencing in late pregnancy. Expectant mothers in their third trimester of pregnancy will be randomised into one of two groups: control group - accessing standard care; or the BLISS (intervention) group - offered BLISS advice in addition to accessing standard care. The study will consist of a 12-month intervention phase with the main outcomes at 12 months of age and a planned follow up at 2 years of age.

The study has been approved by the Lower South Regional Ethics Committee (LRS/11/09/037) and is registered with the Australian New Zealand Clinical Trials Registry ACTRN12612001133820. Written informed consent will be obtained from all participants before randomisation.

### Participants and recruitment

All pregnant women booked into the Queen Mary Maternity Unit, Dunedin Hospital (Dunedin, New Zealand), will be invited to participate in the BLISS study during the third trimester of pregnancy. There are no other birthing facilities in Dunedin (population 120,000) and the number of home births is <3 %. Each woman will receive a letter that acknowledges their booking into the maternity unit and provides them with initial information about the study. Women requesting home births will be given similar information regarding the study from their Lead Maternity Carer (LMC; all mothers in New Zealand choose a LMC, usually a midwife, who is responsible for their pregnancy-related health care from pregnancy to approximately 6 weeks after birth). Just before 28 weeks gestation, the prospective participant’s LMC will be contacted to ensure that invitation letters are not sent to women who have miscarried. At 28 weeks gestation, the prospective participant will receive a letter inviting them to take part in the study. This letter contains an opt-out phone number for an answerphone where the woman can leave a message advising if they do not wish to participate. Research staff will contact women who do not opt-out within 2 weeks to establish eligibility, explain the purpose of the study, answer any questions and if they are interested in participating, organise a time for an individual meeting so that the woman can give written informed consent to participate.

#### Inclusion criteria

Women will be eligible to participate if they: book into the birthing unit at Queen Mary Maternity Hospital before 34 weeks gestation (those women who have chosen a home birth will be considered eligible if their midwife notifies the study before 34 weeks gestation); speak English or Te Reo Māori (the official language of the indigenous people of New Zealand); plan to live in the Dunedin, New Zealand, area until their child is at least 2 years of age; and are 16 years of age or older.

#### Exclusion criteria

After birth, women will be excluded if their infant is born before 37 weeks gestation; or if a congenital abnormality, physical condition, or intellectual disability, which is likely to affect the infant’s feeding or growth is identified.

### Sample size

Reference data for sample size calculations for our primary aim were obtained from our ongoing Prevention of Overweight in Infancy study for which we have data on growth from 0 to 12 months in 491 participants [37]. Using a mean (standard deviation) of 17.3 kg/m^2^ (1.4) and a correlation between repeated measures (BMI at 6 and 12 months) of 0.78, our study has 80 % power at the 5 % level of significance to detect a difference in BMI of 0.40 kg/m^2^ (25 % of a standard deviation) with 85 infants in each group. Comparable differences have been observed in other obesity prevention initiatives during infancy [38].

Sample sizes for selected secondary objectives for which appropriate data were available (power 80 %, significance 5 %) range from 63 to 84 as shown in Table 2.

**Table 2:** Sample size calculations for secondary outcomes

	Reference data	Source of reference data	Difference detected^a^	Number needed per group
	Mean (SD)			
Energy self-regulation scale	3.9 (0.8)	[45]	0.4	63
Plasma ferritin (μg/L)	16.0 (0.6)	[65]	5.0	84

^a^ The difference that could be detected with 80 % power and a significance level of 5 %

We will recruit 200 participants, which allows for a 15 % drop-out for the primary objective and provides sufficient participants for the secondary objectives listed in Table 2.

### Randomisation

The participants will be randomised into one of the two study groups using numbers from random length blocks, after stratification for parity (including the current pregnancy: 1 child vs >1 child) and education (non tertiary vs tertiary), as these may affect responsiveness to the intervention. Research staff will open the next consecutive opaque, pre-sealed envelope in the stratum to which the participant belongs and inform the participant which group they have been assigned to. All outcome assessment data will be collected by research staff blinded to group allocation.

### Study groups

All participants will receive standard Well Child care (a nationally funded health care programme for children under 5 years of age [39]) from the LMC and then Well Child agency of their choice. These free home and clinic visits provide advice on feeding, sleep and safety; and assess growth and development, hearing, vision and wellness for all children within New Zealand. Visits are typically scheduled for: birth, 1 week, 2–4 weeks, and 4–6 weeks (provided by an LMC – typically a community-based midwife); and 8–10 weeks, 3–4 months, 5–7 months, 9–12 months, 15–18 months, and 2–3 years (typically provided by a Well Child nurse) [39].

#### Control group

Participants randomised to the control group will receive standard Well Child care (as described above) from the providers of their choice and no additional intervention.

#### BLISS group

Participants randomised to the BLISS group will receive standard Well Child care (as described above) from the providers of their choice, as well as additional parent contacts for support and education from before birth to 9 months of age delivered by the BLISS study. The intervention will be delivered by an experienced lactation consultant and trained research staff who will be supervised by a multidisciplinary team (dietitian, paediatrician, speech-language therapist) throughout the study. The intervention has three key components:

**Professional lactation consultant service** (third trimester of pregnancy to 6 months of age) - There will be at least five contacts with an International Board Certified Lactation Consultant (IBCLC):

- An anticipatory guidance group session before birth (at approximately 34–35 weeks gestation) to discuss breastfeeding (benefits, challenges and developing a “breastfeeding plan”), explain the nature of the free support service on offer until their infant is 6 months of age and introduce the concept of Baby-Led Introduction to SolidS.
- A home visit in the first week after the mother returns home from hospital, or during the first week following a planned homebirth; a support phone call and offer of a home visit at 3–4 weeks; a home visit at 3–4 months; and a phone call at 5 months of age, to provide support and education around breastfeeding (or formula feeding if requested), and to assess how the recommended approach of milk only until 6 months is going. Support will include encouraging: exclusive breastfeeding to 6 months, breastfeeding to at least 12 months and delaying the introduction of complementary foods until 6 months of age.
- The lactation consultant will also be available to supply additional support when requested by the participant until her infant is 6 months of age. This will involve providing specific individualized advice to address problems with breastfeeding (or formula feeding) via extra home visit(s), phone or email contact. In our earlier Prevention of Overweight in Infancy study [37], this additional support was utilized by 36 % of families (Davies, personal communication).

**BLISS advice** (5.5–9 months of age) – There will be at least three contacts with a trained researcher.

- A home visit at 5.5, 7 and 9 months of age providing individualized advice and support for the introduction of complementary foods using the BLISS approach. Parent participants will be advised that they must not start BLISS until their infant is 180 days (i.e. 6 months of age). Research staff will encourage responsive feeding [40], ensuring that: the infant is not distracted while eating, and caregivers pay attention to the infant’s hunger and satiety cues and respond to the infant promptly and supportively. Parents will be encouraged to offer “easy” foods and more frequent milk feeds during both illness and recovery [1]. A range of resources will be given to participants explaining how to follow BLISS and providing age-appropriate family recipes (see below).
- The researcher will also be available to provide additional support when requested by the participant.

**BLISS resources** (third trimester of pregnancy to 9 months of age) - A range of resources developed and pretested for the purposes of this study will be provided to the participants, including information about the BLISS study, recipe books, everyday food lists and safety information [41]. These resources follow the philosophy of BLW but also address the three key concerns that some health professionals have expressed about BLW [18]: inadequate iron intake, choking and growth faltering. All resources have been developed in conjunction with a paediatric speech-language therapist to address concerns about choking. In particular, the resources encourage parents to:

- Test foods before they are offered to ensure they are soft enough to mash with the tongue on the roof of the mouth (or are large and fibrous enough that small pieces do not break off when sucked and chewed, e.g., strips of meat).
- Avoid offering foods that form a crumb in the mouth.
- Make sure that the foods offered are at least as long as the child’s fist, on at least one side of the food.
- Make sure the infant is always sitting upright when they are eating – never leaning backwards.
- Always have an adult with the child when they are eating.
- Never put whole foods into the infant’s mouth – the infant must do this at their own pace and under their own control.

Parents will be encouraged to offer three food types at each meal:

1. An iron-rich food (e.g., red meat, iron fortified infant cereal).
2. An energy-rich food.
3. A food such as a fruit or vegetable.

**Fig. 2:**
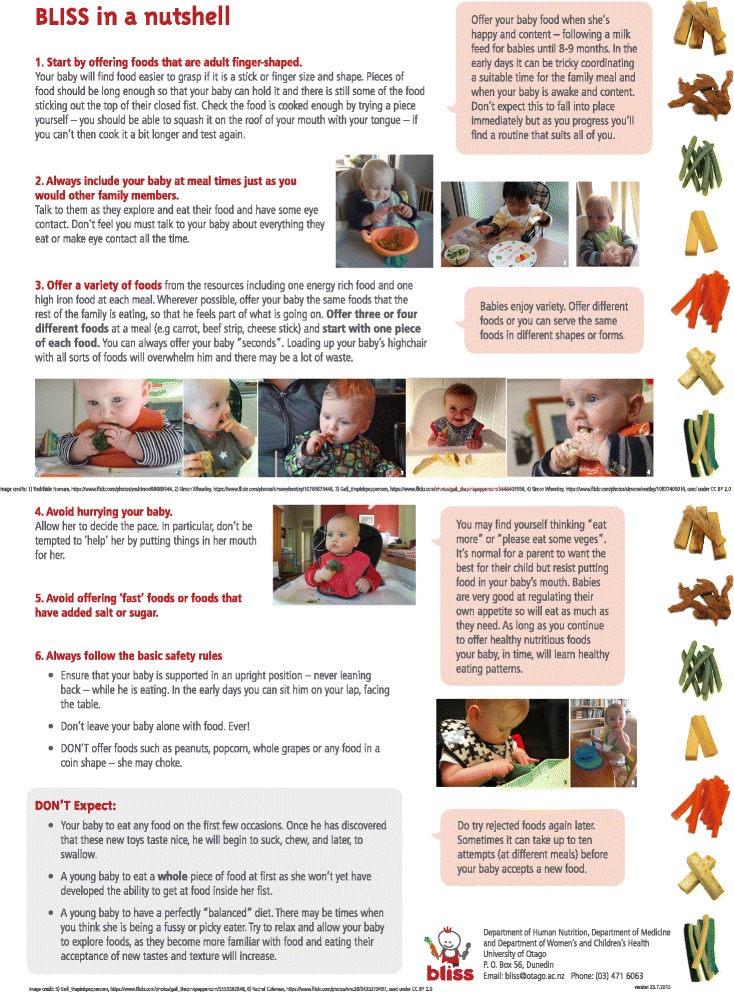
BLISS in a Nutshell

A range of resources will be used at the different visits: ante-natal (*n* = 1), 3–4 months (*n* = 1), 5.5 months (*n* = 6), 7 months (*n* = 2) and 9 months (*n* = 1). Figure 2 shows an example of a resource – the “BLISS in a nutshell” resource which is used (at the 5.5 month visit) to provide an overall summary of the BLISS approach to complementary feeding from 6 months of age and which parents are encouraged to use to help explain BLISS to their infant’s other carers.

### Adherence

Adherence to infant self-feeding will be determined using data provided in the 3-day diet record on who fed the child each food (child, parent, or both) for 3 days over a period of a month. This will provide very detailed data on adherence collected in “real-time”. However, it is likely that not all participants will complete all 3 days of diet recording. For this reason, we will also use a brief (5–10 min) feeding questionnaire at 2, 4, 6, 7, 8, 9 and 12 months to assess adherence to self-feeding. Adherence to the recommendation to exclusively breastfeed to 6 months, and to introduce complementary foods at 6 months, will also be determined using the brief feeding questionnaires at 2, 4, 6, 7, 8, 9 and 12 months.

### Outcome measures

The timing of the outcome measures is presented in Table 3. The primary outcome measure is BMI-for-age z-score (calculated using body weight and length). Secondary outcome measures include: energy self-regulation, iron and zinc intake and status, diet quality, parental feeding behaviour, overall acceptability, choking, and growth faltering.

**Table 3:**
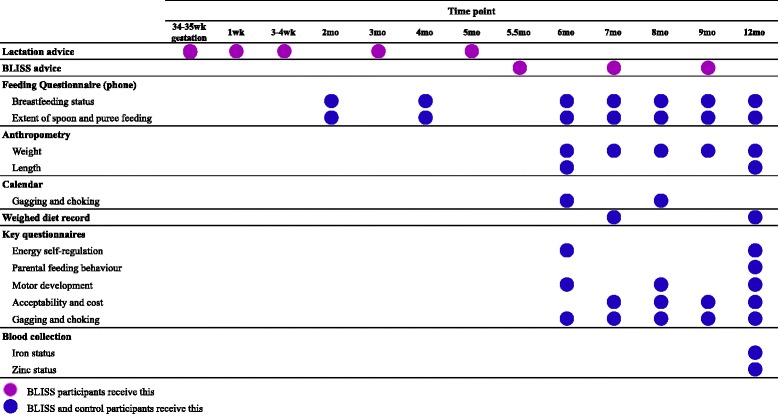
Interventions and outcome measures at specified time points

#### Anthropometric measures

Birth weight will be accessed from hospital records. Length will be measured at 6 and 12 months and body weight at 6, 7, 8, 9 and 12 months of age by trained anthropometrists, using standard paediatric anthropometric techniques [42]. All infant participants will wear a standard nappy of known weight which is provided to the parent and a singlet top. The weight of both items of clothing will be subtracted from the reported body weight before analysis. Body weight will be measured and recorded to the nearest 0.1 kg using digital scales (Seca, Model 334, Hamburg, Germany), which will be calibrated (using a 1 kg or 5 kg calibration weight) prior to each measurement session. Recumbent length will be measured to the nearest 0.1 cm using a portable length board (Harlow Healthcare Rollameter, UK) which will be calibrated (using a 90 cm calibration rod) prior to each measurement session.

Body weight and length measurements will be taken in duplicate and if the second measurement differs by more than 0.1 kg for weight and 0.7 cm for length, a third measure will be taken [42]. An average of the measures will be recorded (where there are three measurements taken, the two closest will be averaged; where the three measures are equidistant the median value will be used). The following will then be calculated: BMI and BMI-for-age z-score at 6 and 12 months of age, and weight-for-age z-score at 6, 7, 8, 9 and 12 months of age, using the WHO child growth standards [43].

Repeated body weight assessment of infants from 6 to 12 months (monthly from 6 to 9 months) will be used to identify growth faltering. Any infant identified as possibly growth faltering (defined as either (a) weight not having increased since the previous measurement, or (b) the difference in weight-for-age z-score between this measurement and the previous one (or the measurement at 6 months) being more negative than -1) will be referred to a paediatrician for further assessment. Growth faltering will be defined (using the WHO growth charts) as ‘a weight deceleration crossing more than two major centile lines, where the major centile lines are 2/3rds of a standard deviation apart’.

#### Questionnaires

A self-administered baseline questionnaire will collect socio-demographic information such as ethnicity, maternal and paternal education, and New Zealand Deprivation Index 2013 score, an indicator of the level of household deprivation [44].

A questionnaire will be completed by adult participants when their infant is 6 and 12 months of age which will assess the infant’s energy self-regulation using an 8-item scale created by Tan et al. [45]. At 6, 7, 8, 9 and 12 months, questions regarding cup or bottle emptying will be used as an indicator of self-regulation of milk intake for those who are offered infant formula or expressed breast milk [46].

Infant temperament will be assessed at 6 months of age using the revised infant temperament questionnaire [47]. The ‘Ages and Stages’ questionnaire [48] will be used in questionnaires at 6, 8 and 12 months of age to assess fine and gross motor skills. Parental feeding behaviour and perception of “picky eating” will be assessed when the infant is 12 months of age [49]. Eating behaviour will also be assessed at 12 months using the Children’s Eating Behaviour Questionnaire by Wardle et al. [50].

Brief feeding questionnaires will be administered at 7, 8, 9 and 12 months of age to assess a range of issues including the acceptability to parents of the complementary feeding approach used. Acceptability will focus particularly on “mess”, convenience, cost and the extent to which the approach “suits you as a parent”. At 8 and 9 months, the primary carer will also be asked about their perception of their partner’s attitude to the complementary feeding approach used.

#### Choking

Questions on gagging and choking, including frequency and a description of the cause and outcome of the most serious choking event in the past month, will be asked in questionnaires at 6, 7, 8, 9 and 12 months of age.

Calendars will also be distributed to parents at 6 and 8 months of age and parents will be asked to indicate on the calendar, each day for a month, whether the infant has gagged or choked that day. Education will be provided on the difference between gagging and choking to help parents distinguish between them. If a choking episode occurs, further information will be requested including the food or drink involved, the form of the food or drink and how the choking episode was resolved.

#### Dietary assessment

The parent will complete a 3-day weighed diet record for the infant at 7 and 12 months of age. Dietary scales accurate to ±1 g (Salter Electronic, Salter Housewares Ltd. Tonbridge, UK) will be used to measure all food and drink consumed by the infant on three randomly assigned non-consecutive days of the week (2 week days and 1 weekend day) over a 3-week period. Each day of the week will be represented an approximately equal number of times among participants to control for day-of-the-week effects. Parents will receive detailed oral and written instructions from trained research staff on how to complete the 3-day diet record.

The diet record will have four key components: (a) the diet record, where information will be recorded regarding the time of the day, type and brand of the food or drink, preparation method, weight of the food or drink, consistency of the food or drink (puréed, mashed, diced or whole), who fed the child (parent, child or both) and the total weight and estimated proportions of leftover food or drink; (b) a description of any recipes used, including the raw amounts of ingredients, the cooking method and proportion of the total recipe fed to the child; (c) an “end of day questionnaire”, which will determine whether this was a typical eating day for the child and how the meals compared to those consumed by the rest of the family; and (d) whether the child had any iron or zinc containing supplements, including type, brand and amount taken.

On completion of the 3-day diet record, a researcher will check the record for omissions and clarify these with the parent. All diet records will be entered into the dietary analysis software programme Kai-culator (University of Otago, New Zealand) for analysis. Kai-culator uses the New Zealand Food Composition Database, FOODfiles [51]; nutrient data for commonly consumed recipes collated in the 2008/09 New Zealand Adult Nutrition Survey [52]; and nutrient data for commercial infant foods collated by the research team [53].

The diet records will be used to determine mean daily intakes of nutrients and food components including: energy, protein, fat, carbohydrate, iron, zinc and phytate; phytate-to-zinc and phytate-to-iron molar ratios [54]; grams of red meat (beef and lamb) and all flesh foods (red meat, non-red meat, poultry and fish); and iron and zinc from different food sources.

#### Biochemical assessment

At 12 months of age a non-fasting venous blood sample will be taken from an antecubital vein between 8:30 and 11:30 am. A questionnaire will be completed 24 h before the blood test appointment to determine any recent illness which may affect blood analyses. If the infant is unwell (presence of fever, diarrhoea or vomiting), the blood sample collection will be delayed for 14 days.

Parents will be asked to:

- Give their infant a milk feed and stop feeding exactly 90 min prior to the blood test appointment [55].
- Not give their infant any other food or fluid (except water) until after the blood test appointment.
- Apply a local anaesthetic, Ametop gel (Smith & Nephew Ltd., Auckland, New Zealand), 1–4 h prior to the blood test appointment. The gel is to be applied to the inside elbow crease of both arms to numb the phlebotomy site, and so that if the initial attempt is unsuccessful, the blood sample can be collected from the other arm.

A trained phlebotomist will collect one peripheral venipuncture blood sample (7.5 mL) from each infant participant into a trace element-free lithium heparin anticoagulated tube (Sarstedt S-Monovette, Nümbrecht, Germany). Blood samples will be refrigerated immediately after collection and for no longer than 2 hours before centrifuging at 2500 × g for 10 min. Before centrifuging, 1 mL of whole blood will be removed for analysis of complete blood count and plasma ferritin. Aliquots of plasma will then be stored at ^−^80 °C until subsequent analysis of soluble transferrin receptor (sTfR), C-reactive protein (CRP), α-1 acid glycoprotein (AGP) and plasma zinc. A Cobas C311 automatic electronic analyzer (Roche, New Zealand) will be used to determine sTfR, CRP and AGP concentrations in the Department of Human Nutrition Trace Element Laboratory (University of Otago, New Zealand).

Complete blood count will be determined using a Sysmex XE 5000 automatic electronic analyzer (Kobe, Japan) and plasma ferritin concentration using a Cobas 8000 unit e 602 (Roche, United States of America) on the day of blood collection by Southern Community Laboratories Ltd. (Dunedin, New Zealand).

Any infant with iron results that are of concern (haemoglobin ≤105 g/L and/or plasma ferritin ≤15 ug/L) will be referred to their general practitioner for treatment but will remain in the study so they can be included in the intention to treat analysis.

The International Zinc Nutrition Consultative Group (IZiNCG) protocol for measuring plasma zinc concentration will be followed [56] ensuring that trace element free techniques are used throughout – from sample collection to analysis. Plasma zinc concentration will be determined using flame atomic absorption spectrophotometry (ContraAA 700, Analytik Jena, Germany) in the Department of Human Nutrition Trace Element Laboratory (University of Otago, New Zealand).

A CRP of >5 mg/L will be used as an index of acute inflammation and an α-1 acid glycoprotein concentration of >1 g/L as an index of chronic inflammation [57]. Soluble transferrin receptor will be converted to be equivalent with the Flowers assay using the following equation: Flowers sTfR = 1.5 × Roche sTfR + 0.35 mg/L [58] then body iron will be calculated as the log ratio of sTfR to plasma ferritin concentration [58]. Iron deficiency will be defined as a body iron concentration <0 mg/kg, and iron deficiency anaemia as iron deficiency and haemoglobin <110 g/L. Low iron stores will be defined as body iron ≥0 mg/kg and serum ferritin concentration <12 μg/L. A plasma zinc <9.9 μmol/L will be used to define zinc deficiency [56].

### Adverse events

The study will identify and monitor adverse events (defined as any untoward or unfavourable medical occurrence in a participant, including any abnormal sign, symptom, or disease, temporally associated with the participant’s participation in the research, whether or not it is considered to be related to the participant’s participation in the study) and “serious adverse events” (defined as any adverse event temporally associated with the participant’s participation in the BLISS study that results in death, is life-threatening, requires inpatient hospitalization, results in a persistent or significant disability or incapacity, any other adverse event that, based upon appropriate medical judgment, may jeopardize the participant’s health and may require medical or surgical intervention to prevent one of the other outcomes listed) [59].

Specific arrangements will be in place for immediate investigation and referral to the study’s paediatric clinicians of choking episodes (reported directly by the participant, or where the involvement of a health professional for a choking incident is reported in the Calendar or in study questionnaires) and growth faltering (identified during the 6, 7, 8, 9 and 12 month anthropometric measurement sessions).

Care will also be taken to identify and investigate: (a) multiple occurrences of the same adverse event in the same participant, (b) occurrence of different adverse events in the same participant, (c) occurrence of the same adverse event in multiple participants.

### Quality control

Measures will be put in place during the course of the study to ensure that the information provided to participants is standardized, that data collected are of high quality and that data collection is as complete as possible.

- **Standard operating procedures** – Detailed protocols will be developed and used for all study-related tasks including participant contacts and data management tasks.
- **Observed interviews** – Research staff will be observed by the investigators of the study twice yearly to ensure that the standard operating procedures are being followed.
- **Data audits** - Every 3 months a data audit will be conducted to check for completeness of collected data.
- **Technical error of measurement –** Inter-evaluator technical error of measurement (TEM) will be determined for all research staff who are responsible for making anthropometric measurements, after initial training and then annually. The TEM will be determined by repeated anthropometric measurements on a separate sample of 5–10 infants [60].
- **Checking of weighed diet records** – Diet records will be checked when they are received. If any data are missing or unclear the participant will be contacted for clarification. After the diet records have been entered in Kai-culator, a New Zealand Registered Dietitian will check each diet record and correct any errors made in the initial calculation and entry of the record, and ensure consistency in the data entry decisions.
- **Biomarkers** ─ The precision of the biochemical assays will be checked using a pooled plasma sample and their accuracy via the use of certified reference materials or manufacturer’s controls, where appropriate.

### Follow-up at 24 months of age

Participants will be followed up when the child participant is 24 months of age. The follow up will consist of anthropometric measurements, a 3-day diet record and a comprehensive questionnaire assessing most of the variables outlined previously (energy self-regulation, parental feeding practices, infant fine and gross motor skills, perception of “picky eating” and acceptability of the complementary feeding approach - all as described above).

### Statistical analysis

The initial analyses will be conducted using intention to treat. The primary analysis will determine whether BLISS results in differences in BMI-for-age z-score at 12 months of age (i.e. at the end of the intervention) and 24 months (i.e. at the end of the planned follow up) of age. Regression analyses will be used to analyze secondary outcomes including iron and zinc intake and status. All plasma zinc analyses will control for time of day, fasting status and time of last food/drink. We will consider adjusting plasma ferritin and zinc concentrations where there is evidence of infection or inflammation, indicated by elevated C-reactive protein or α-1 acid glycoprotein [57, 61]. Statistical significance will be defined as *P* <0.05.

Subsequent to this we will also do a per protocol analysis in order to identify any differences to the intention to treat analysis, which may occur because of differential compliance with the research protocol.

## Discussion

With the increasing popularity of BLW amongst parents and interest from health professionals who need to provide evidence-based advice to parents on how to safely introduce complementary foods, there is an urgent need for data on the potential benefits and risks of a baby-led approach to complementary feeding. To our knowledge, this is the first randomised controlled trial of a baby-led approach to complementary feeding so it will generate much new evidence in the area of infant feeding, particularly during the second 6 months of life. While some governments are already modifying their advice on the most appropriate texture for first foods [13, 14, 62], countries such as New Zealand are waiting for evidence from well-designed randomised controlled trials.

The primary aim of this intervention study is to determine whether a baby-led approach to complementary feeding can improve body weight status at 12 months of age. With childhood obesity rates growing worldwide [63], the study will provide insight into the development and establishment of eating patterns from the start of the complementary feeding period and their effect on body weight during infancy and early childhood. We hypothesize that self-feeding throughout the complementary feeding period may help infants maintain and develop the energy self-regulation skills they have developed while exclusively milk-feeding [64] and that this behaviour will continue into later childhood.

The BLISS study will also investigate the potential negative effects of BLW and determine whether this modified approach, BLISS, is able to prevent iron deficiency and minimize any risk of choking or growth faltering. This information is extremely important both to enable health professionals to advise parents who would like to follow a baby-led approach to complementary feeding and to advise policy-makers on whether this approach should replace current advice on complementary feeding for parents in general.

## Abbreviations

BLISS: Baby-Led Introduction to SolidS
BLW: Baby-Led Weaning
BMI: body mass index
CRP: C-reactive protein
IBCLC: International Board Certified Lactation Consultant
IZiNCG: International Zinc Nutrition Consultative Group
LMC: lead maternity carer
sTfR: soluble transferrin receptor
UK: United Kingdom
WHO: World Health Organization

## References

[1] World Health Organization (WHO) (2004). Guiding principles of complementary feeding of the breastfed child.

[2] World Health Organization (WHO) (2005). Guiding principles for feeding non-breastfed children 6–24 months of age.

[3] World Health Organization (WHO) (2009). Infant and young child feeding: model chapter for textbooks for medical students and allied health professionals.

[4] Naylor AJ, Morrow AL. Developmental readiness of normal full term infants to progress from exclusive breastfeeding to the introduction of complementary foods: Reviews of the relevant literature concerning infant immunologic, gastrointestinal, oral motor and maternal reproductive and lactational development. Washington: The Linkages Project and Wellstart International; 2001.

[5] Agostoni C, Decsi T, Fewtrell M, Goulet O, Kolacek S, Koletzko B (2008). Complementary feeding: a commentary by the ESPGHAN committee on nutrition. J Pediatr Gastroenterol Nutr.

[6] Prescott SL, Smith P, Tang M, Palmer DJ, Sinn J, Huntley SJ (2008). The importance of early complementary feeding in the development of oral tolerance: Concerns and controversies. Pediatr Allergy Immunol.

[7] Fewtrell MS, Lucas A, Morgan JB (2003). Factors associated with weaning in full term and preterm infants. Arch Dis Child Fetal Neonatal Ed.

[8] Ministry of Health (MOH) (2008). Food and nutrition guidelines for healthy infants and toddlers (Aged 0–2): a background paper.

[9] United States Department of Agriculture (2009). Infant nutrition and feeding. Chapter 5: Complementary foods.

[10] National Health and Medical Research Council (NHMRC), Department of Health and Ageing, Australian Government (2012). Infant feeding guidelines: information for health workers.

[11] Wright CM, Cameron K, Tsiaka M (2011). Is baby-led weaning feasible? When do babies first reach out for and eat finger foods?. Matern Child Nutr.

[12] Rapley G (2011). Baby-led weaning: transitioning to solid foods at the baby’s own pace. Community Pract.

[13] Department of Health United Kingdom (UK) (2009). Birth to five (Updated June 2010).

[14] Health Canada. Nutrition for healthy term infants: recommendations from six to 24 months. Health Canada; 2014. http://www.hc-sc.gc.ca/fn-an/nutrition/infant-nourisson/recom/recom-6-24-months-6-24-mois-eng.php 10.3148/73.4.2012.204 23217450

[15] Cameron SL, Heath ALM, Taylor RW (2012). How feasible is baby-led weaning as an approach to infant feeding? A review of the evidence. Nutrients.

[16] Arden MA, Abbott RL (2014). Experiences of baby-led weaning: trust, control and renegotiation. Maternal Child Nutr.

[17] Townsend E, Pitchford NJ (2012). Baby knows best? The impact of weaning style on food preferences and body mass index in early childhood in a case-controlled sample. BMJ Open.

[18] Cameron SL, Heath ALM, Taylor RW (2012). Healthcare professionals’ and mothers’ knowledge of, attitudes to and experiences with, Baby-Led Weaning: a content analysis study. BMJ Open.

[19] Cameron SL, Taylor RW, Heath ALM (2013). Parent-led or baby-led? Associations between complementary feeding practices and health-related behaviours in a survey of New Zealand families. BMJ Open.

[20] Brown A, Lee M (2011). A descriptive study investigating the use and nature of baby-led weaning in a UK sample of mothers. Maternal Child Nutr.

[21] Brown A, Lee M (2011). Maternal control of child feeding during the weaning period: differences between mothers following a baby-led or standard weaning approach. Matern Child Health J.

[22] Rowan H, Harris C (2012). Baby-led weaning and the family diet. A pilot study. Appetite.

[23] Ministry of Health (MOH). Baby-Led Weaning – Ministry position statement. Ministry of Health; 2012. http://www.health.govt.nz/our-work/preventative-health-wellness/nutrition/baby-led-weaning-ministry-position-statement. Accessed 12 May 2014.

[24] Rapley G, Murkett T (2008). Baby-Led Weaning.

[25] Schwartz C, Scholtens PA, Lalanne A, Weenen H (2011). Development of healthy eating habits early in life. Review of recent evidence and selected guidelines. Appetite.

[26] Brown A, Lee M (2013). An exploration of experiences of mothers following a baby-led weaning style: developmental readiness for complementary foods. Maternal Child Nutr.

[27] Gross RS, Mendelsohn AL, Fierman AH, Messito MJ (2011). Maternal controlling feeding styles during early infancy. Clin Pediatr (Phila).

[28] Brown A, Lee MD (2013). Early influences on child satiety-responsiveness: the role of weaning style. Pediatr Obes.

[29] Faith MS, Scanlon KS, Birch LL, Francis LA, Sherry B (2004). Parent–child feeding strategies and their relationships to child eating and weight status. Obes Res.

[30] Brown A, Lee M (2011). Maternal child-feeding style during the weaning period: Association with infant weight and maternal eating style. Eat Behav.

[31] Carruth BR, Skinner JD (2002). Feeding behaviors and other motor development in healthy children (2-24 months). J Am Coll Nutr.

[32] Domellöf M, Braegger C, Campoy C, Colomb V, Decsi T, Fewtrell M (2014). Iron requirements of infants and toddlers. J Pediatr Gastroenterol Nutr.

[33] Walter T, Dallman PR, Pizarro F, Velozo L, Pena G, Bartholmey SJ (1993). Effectiveness of iron-fortified infant cereal in prevention of iron-deficiency anemia. Pediatrics.

[34] Szymlek-Gay EA, Ferguson EL, Heath ALM, Gray AR, Gibson RS (2009). Food-based strategies improve iron status in toddlers: a randomized controlled trial. Am J Clin Nutr.

[35] Heinig MJ, Nommsen LA, Peerson JM, Lönnerdal B, Dewey KG (1993). Energy and protein intakes of breast-fed and formula-fed infants during the first year of life and their association with growth velocity: the DARLING study. Am J Clin Nutr.

[36] Gibson R, Heath AL (2011). Population groups at risk of zinc deficiency in Australia and New Zealand. Nutr Diet.

[37] Taylor BJ, Heath ALM, Galland BC, Gray AR, Lawrence JA, Sayers RM (2011). Prevention of Overweight in Infancy (POI.nz) study: a randomised controlled trial of sleep, food and activity interventions for preventing overweight from birth. BMC Public Health.

[38] Wen LM, Baur LA, Simpson JM, Rissel C, Wardle K, Flood VM (2012). Effectiveness of home based early intervention on children’s BMI at age 2: randomised controlled trial. BMJ.

[39] Ministry of Health (MOH). Well Child/Tamariki Ora*.* Ministry of Health; 2014. http://www.health.govt.nz/your-health/services-and-support/health-care-services/well-child-tamariki-ora. Accessed 11 May 2014.

[40] Black MM, Aboud FE (2011). Responsive feeding is embedded in a theoretical framework of responsive parenting. J Nutr.

[41] Cameron SL. Is Baby-led Weaning a feasible method for introducing complementary foods to infants in New Zealand? PhD Thesis. University of Otago, Department of Human Nutrition; 2014.

[42] de Onis M, Onyango AW, Van den Broeck J, Chumlea WC, Martorell R (2004). Measurement and standardization protocols for anthropometry used in the construction of a new international growth reference. Food Nutr Bull.

[43] World Health Organization (WHO) (2009). WHO Child growth standards and the identification of severe acute malnutrition in infants and children.

[44] Atkinson J, Salmond C, Crampton P (2014). NZDep2013 index of deprivation user’s manual.

[45] Tan CC, Holub SC (2011). Children’s self-regulation in eating: Associations with inhibitory control and parents’ feeding behavior. J Pediatr Psychol.

[46] Li R, Fein SB, Grummer-Strawn LM (2010). Do infants fed from bottles lack self-regulation of milk intake compared with directly breastfed infants?. Pediatrics.

[47] Gartstein MA, Rothbart MK (2003). Studying infant temperament via the revised infant behavior questionnaire. Infant Behav Dev.

[48] Ages and Stages Questionnaire (ASQ-3). Brookes Publishing Co. 2015. www.agesandstages.com/asq-products/asq-3/. Accessed 3 July 2014.

[49] Horodynski MA, Stommel M, Brophy-Herb H, Xie Y, Weatherspoon L (2010). Low-income African American and Non-Hispanic white mothers’ self-efficacy, ‘picky eater’ perception, and toddler fruit and vegetable consumption. Public Health Nurs.

[50] Wardle J, Guthrie CA, Sanderson S, Rapoport L (2001). Development of the children’s eating behaviour questionnaire. J Child Psychol Psychiat.

[51] Ministry of Health (MOH), New Zealand Institute for Plant and Food Research Limited. New Zealand food composition database (FOODfiles)*.* 2013. www.foodcomposition.co.nz/foodfiles. Accessed 4 July 2014.

[52] Ministry of Health (MOH), University of Otago (2011). Methodology report for the 2008/09 New Zealand adult nutrition survey.

[53] Clouston A. The role of commercial processed baby foods in the diets of New Zealand toddlers. MDiet Thesis. University of Otago, Department of Human Nutrition; 2014.

[54] Hotz C, Brown KH (2004). International Zinc Nutrition Consultative Group (IZiNCG) technical document. Food Nutr Bull.

[55] Arsenault JE, Wuehler SE, de Romaña DL, Penny ME, Sempertegui F, Brown KH (2011). The time of day and the interval since previous meal are associated with plasma zinc concentrations and affect estimated risk of zinc deficiency in young children in Peru and Ecuador. Eur J Clin Nutr.

[56] International Zinc Nutrition Consultative Group (IZiNCG) (2007). IZiNCG technical brief 2: assessing population zinc status with serum zinc concentration.

[57] Thurnham DI, McCabe LD, Haldar S, Wieringa FT, Northrop-Clewes CA, McCabe GP (2010). Adjusting plasma ferritin concentrations to remove the effects of subclinical inflammation in the assessment of iron deficiency: a meta-analysis. Am J Clin Nutr.

[58] Cogswell ME, Looker AC, Pfeiffer CM, Cook JD, Lacher DA, Beard JL (2009). Assessment of iron deficiency in US preschool children and nonpregnant females of childbearing age: National Health and Nutrition Examination Survey 2003-2006. Am J Clin Nutr.

[59] U.S. Department of Health and Human Services. Guidance on reviewing and reporting unanticipated problems involving risks to subjects or others and adverse events. http://www.hhs.gov/ohrp/policy/advevntguid.html. Accessed 24 October 2014.

[60] World Health Organization (WHO) Multicentre Growth Reference Study Group (2006). Reliability of anthropometric measurements in the WHO Multicentre Growth Reference Study. Acta Paediatr.

[61] Brown KH, Lanata CF, Yuen ML, Peerson JM, Butron B, Lönnerdal B (1993). Potential magnitude of the misclassification of a population’s trace element status due to infection: example from a survey of young Peruvian children. Am J Clin Nutr.

[62] Department of Health United Kingdom (UK) (2013). Birth to five. Chapter 3. Introducing your baby to solid food.

[63] Dinsdale H, Ridler C, Ells L (2011). NOO National Obesity Observatory: a simple guide to classifying body mass index in children.

[64] Dewey KG, Lönnerdal B (1986). Infant self-regulation of breast milk intake. Acta Paediatr Scand.

[65] Soh P, Ferguson EL, McKenzie JE, Homs M, Gibson RS (2004). Iron deficiency and risk factors for lower iron stores in 6–24-month-old New Zealanders. Eur J Clin Nutr.

